# Phytoremediation potential of invasive plant species for potentially toxic elements along the Sava River upstream

**DOI:** 10.1016/j.heliyon.2024.e33798

**Published:** 2024-07-02

**Authors:** Zorana Miletić, Snežana Jarić, Milica Jonjev, Miroslava Mitrović, Dragana Pavlović, Marija Matić, Pavle Pavlović

**Affiliations:** Department of Ecology, Institute for Biological Research ‘Siniša Stanković’, University of Belgrade, Bulevar despota Stefana 142, Belgrade, Serbia

**Keywords:** *Solidago canadensis*, *Reynoutria japonica*, *Impatiens glandulifera*, Potentially toxic elements, Heavy metals, Trace elements

## Abstract

Invasive plant species (IPS) have many characteristics that are necessary for successful phytoremediation and the accumulation of large amounts of potentially toxic elements (PTEs). The most common IPS from the source of the Sava River are Re*ynoutria japonica, Solidago canadensis* and *Impatiens glandulifera*. Considering that the riparian soils of the Sava River are classified as moderately polluted, this study investigated their enrichment with PTEs (As, B, Cd, Cr, Cu, Ni, Pb, Zn) and the potential for accumulation of these elements in roots and leaves of the most common IPS. The soil and plant samples were prepared using the wet digestion method in CEM Mars 6 microwave oven. The content of PTEs in soil and plant samples was determined by ICP-OES. The results showed a moderate to very high enrichment of Cu in the soils. *Impatiens glandulifera* has the highest uptake capacity in leaves and phytoextraction ability of B, Cd, Cu and Zn. *Reynoutria japonica* has some potential for phytoextraction of Pb, Zn and especially B, while *Solidago canadensis* has potential for phytoextraction of B and Zn, while excluding Cr and Cu. The analyzes have shown that the studied species are not suitable for bioindication of PTEs in the riparian soils of the Sava River. Considering the enrichment of soils with Cu, *Impatiens glandulifera* is the most suitable species for phytoremediation of these elements among the studied species.

## 1 Introduction

Increasing industrial development has led to increased levels of heavy metals and other potentially toxic elements (PTEs) in ecosystems, resulting in various problems in biology, agriculture and human health [1]. Pollution with PTEs pervades ecosystems, especially in industrial areas and near mines [2]. As reported by Hu et al. [3], about twenty million hectares of land worldwide are contaminated with these pollutants.

Considering the harmful effects of PTEs on living organisms, effective measures are needed to mitigate their damage. The physico-chemical method of removing PTEs from soil is destructive, costly and leads to disturbance of agricultural land, resulting in secondary pollution [4]. Phytoremediation, in which plants are used to remove pollutants from various sources, is considered as a cost-effective and efficient method [5]. For effective phytoremediation, plants require certain characteristics such as fast growth, high biomass, indigestibility, robust root systems and the ability to hyperaccumulate contaminants while being tolerant to various environmental stresses. The identification of plants with rapid growth, substantial biomass and robust tolerance is crucial to improve the effectiveness of phytoremediation strategies [6]. However, many plants cannot survive on contaminated sites due to the toxicity of pollutants, making invasive plant species (IPS) more suitable for phytoremediation [7]. Recent studies show that IPS have characteristics that are necessary for for effective phytoremediation [8,9].

IPS can provide unrecognized economic, social and environmental benefits [10] and make a positive contribution to various services [11,12]. In practice, IPS show the desirable properties associated with hyperaccumulators: increased tolerance to pollutants [13], resistance to drought [14] and allelopathic abilities [15]. They play a role in the uptake of toxic metals from the soil via the rhizosphere by immobilizing these metals and significantly reducing their concentrations in the soil [16]. The ability of IPS to remove PTEs also contributes to their successful invasion. Therefore, the prudent use of IPS has become an important concern and a focus of research [6,17].

Numerous studies have emphasized the use of various IPS such as *Calotropis gigantea, Sida cardifolia, Ricinus communis, Spartina alterniflora, Alternanthera philoxeroides* and *Eichhornia crassipes* for phytoremediation [18]. In a Pb-contaminated environment, the invasive weed *Solidago canadensis* was grown alongside the native plant *Kummerowia striata* to evaluate its tolerance. The results showed that *S. canadensis* has a higher accumulation of Pb and effectively binds it in its aboveground tissues [19]. Another study has shown that *Reynoutria japonica* has a high tolerance to Cd, Cr, Pb and Zn [20]. Research on hyperaccumulators among IPS remains crucial despite certain limitations. Investigating their cost-effectiveness and environmental friendliness for phytotechnological innovations is essential [21]. Their application in the treatment of PTEs in soils through phytoremediation is proving to be a sustainable approach for the restoration of invaded forests and agroforestry systems [22].

The Sava River basin provides an exceptional opportunity to study the interplay between natural and man-made factors that influence the chemical dynamics of a river and the invasion of the ecosystem. The presence of PTEs in the riparian environment is a major pollution problem that constantly threatens the health of riparian organisms and, consequently, humans. These metals are highly toxic and persistent and tend to accumulate in sediments and living organisms [23]. So far, the potential of some IPS for bioindication and phytoremediation along the Sava River has been investigated for Li and Sr [24], but not for other PTEs. As the Sava River is a corridor for the IPS dispersal, it is important to investigate the potential of established IPS and to exploit the potential benefits of their presence. According to the results of the SAVA TIES project (Preserving Sava River Basin Habitats through Transnational Management of Invasive Alien Species), the most abundant IPS along the Sava River are *Amorpha fruticosa, Reynoutria japonica* and *Solidago canadensis* [25]. During the field study, we found that the most common IPS from the source of the river to the city of Zagreb (Croatia) are *Reynoutria japonica, Solidago canadensis* and *Impatiens glandulifera*.

The objectives of this study are therefore to: (I) to estimate the extent of contamination of soils with PTEs in the riparian zone of the upper and middle reaches of the Sava River; (II) to determine the physical and chemical properties of soils that could influence the accumulation of PTEs in plants; (III) to measure the content of As, B, Cd, Cr, Cu, Ni, Pb and Zn in the roots and leaves of *Reynoutria japonica, Solidago canadensis* and *Impatiens glandulifera*; (IV) to assess the potential of these species for bioindication and phytoremediation of PTEs in the riparian soils of the Sava River.

## 2 Material and methods

### 2.1 Study area and sampling sites

The Sava catchment area, which covers an area of around 97.700 km^2^, is an important catchment area in south-eastern Europe. Historically, the Sava was of great national importance in the former Yugoslavia and after its disintegration in the early 1990s, it became an internationally recognized river of considerable importance. As the largest drainage and third longest tributary of the Danube, the Sava connects four states and three capitals (Ljubljana, Zagreb and Belgrade). The Sava is considered the northern border of the Balkan Peninsula. It belongs to the catchment area of the Black Sea and is the third longest tributary of the Danube.

**Fig. 1:**
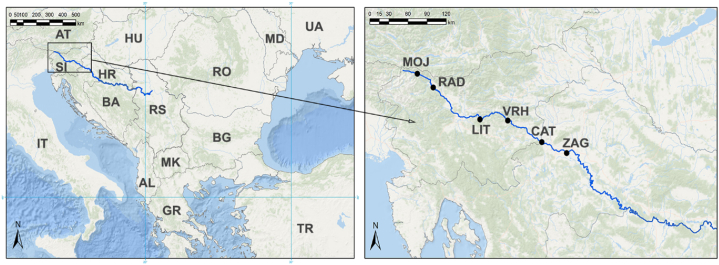
Position of the Sava River on Balkans with marked sampling sites.

Anthropogenic influences within the catchment area include thermal and hydroelectric power plants, oil and gas refineries with their associated pipelines, the metallurgical, chemical and textile industries and mining. These human activities contribute to the environmental pressures observed in the Sava River basin. The study area shows a clear pollution gradient, ranging from relatively low to moderately polluted sites upstream of the city of Zagreb, and the city of Zagreb itself with 1 million inhabitants and a strong industrial presence. These areas are characterized by naturally elevated PTEs with anthropogenic input from mining and industrial activities [26]. The attributes and the position of the sampling sites are presented in Fig. 1 and Table 1. Selection of the sampling sites was made in accordance with the European Commission's FP7 project 'Managing the effects of multiple stressors on aquatic ecosystems under water scarcity' (GLOBAQUA) [27].

**Table 1:** Sampling site properties.

Sampling site	Full name	State	Latitude (N)	Longitude (E)	Population	Climate		Contamination sources
						Average temperature	Total percipitation	
MOJ	Mojstrana	Slovenia	46°27.59ʹ	13°56.41ʹ	1.200	7.9 °C	1.257 mm	PTEs from the weathering of the bedrock
RAD	Radovljica	Slovenia	46°20.37ʹ	14°9.83ʹ	6.000	8.8 °C	1.177 mm	The metal processing industry located upstream
LIT	Litija	Slovenia	46°3.34ʹ	14°49.39ʹ	6.500	10.2 °C	1.210 mm	Abandoned mining activities
VRH	Vrhovo	Slovenia	46°2.72ʹ	15°12.92ʹ	300	10.7 °C	1.114 mm	Dam -hydromorphological alteration of the river
CAT	Čatež	Slovenia	45°51.30ʹ	15°41.67ʹ	320	9.4 °C	1.240 mm	Viticulture and wood processing industry
ZAG	Zagreb	Croatia	45°45.42ʹ	16°2.74ʹ	800.000	11.0 °C	930 mm	PTEs from industrial and urban activities

### 2.2 Soil and plant sampling and preparation

The soil samples were taken in the floodplains, 10–15 m away from the river flow. The samples were taken from a depth of 0–10 cm. For the preparation of composite samples 5 soil samples from the root zone of the studied plant species were pooled at each sampling site. The samples were taken at a depth of 0–10 cm, as the soils at the selected sites in the mountain region are shallow and poorly developed. Moreover, this soil depth is suitable for the analysis of PTEs, considering that PTEs from anthropogenic sources accumulate in the surface layer of the soil [28]. The samples were air dried, ground with a stainless steel grinder (Polimix, Kinematica AG) and sieved through a sieve with an opening of 2 mm. These processed samples were then stored in clean polypropylene bags for subsequent analysis. The samples were dried in an oven (Binder, Tuttlingen, Germany) at 105 °C until a constant weight was achieved.

For plant analysis, 30 g of leaves were taken from the aerial parts of three to five selected individuals and root samples from the rhizosphere. For each species, composite samples were prepared at each sampling site. Sampling was carried out in early September, at the end of the growing season, to ensure maximum PTE accumulation in all plant species. Root and leaf samples were washed with distilled water and dried in an oven at 75 °C until a constant weight was reached. The samples were then ground in a laboratory mill (Polimix, Kinematica AG), sieved through a stainless steel sieve with a mesh size of 1.5 mm and further processed for analysis.

### 2.3 Soil physical and chemical properties analyses

The physical and chemical properties of the soil at the sampling sites were assessed from aggregate samples taken at each sampling site. The granulometric composition was determined using the sedimentation method with a combined pipetting technique in a 0.4 M tetrasodium diphosphate (Na_4_P_2_O_7_) solution (Fisher Chemical, 99.7 % purity) according to the method of Atterberg [29]. The analysis included the measurement of sand (2–0.06 mm), silt (0.06–0.002 mm) and clay particle content (<0.002 mm) as well as classification of soil texture classes based on the texture triangle described by the USDA [30].

The active pH values in water (H_2_O) were measured with a pH meter (WTW, inoLab 7110, Germany) to determine the acidity or alkalinity of the soil. In addition, the organic matter content (%) of the soil was determined using a titration method. In this method, (NH_4_)_2_Fe(SO_4_)_2_ × 6H_2_O (CARLO ERBA Reagents, purity >99.5 %) was used after the samples were digested with a dichromate-sulphuric acid solution (from Potassium dichromate - Fisher Cehmical, purity >99.5 %, and Sulphuric acid 95–97 % - Labexpert UK), following Simakov's modification of the Turin method [31].

### 2.4 PTE content in soil and plant samples

To determine the PTE content of the soil, 0.5 g were digested with aqua regia (a mixture of 3 ml HNO_3_ – 65 % Lach-Ner and 9 ml HCl – 37 % Panreac) using a microwave oven (CEM Mars 6). Optical emission spectrometry by ICP-OES (Spectro Genesis) was used to measure the content of PTEs in the digested samples. The accuracy of these measurements was confirmed by analyzing standard reference material (clay soil - ERM-CC141, IRMM certified by EC-JRC), which showed recoveries between 95 % and 106 %.

For the plants, 0.3 g of plant material was wet digested with a mixture of nitric acid (9 ml 65 % HNO_3_ - Lach-Ner) and hydrogen peroxide (3 ml 30 % H_2_O_2_, CARLO ERBA Reagents) in a microwave oven (CEM Mars 6). The content of elements in the plants was again determined by ICP-OES (Spectro Genesis). To check the accuracy of the results, standard reference material (Beach leaf - BCR-100, IRMM certified by EC-JRC) was analyzed, which showed recovery values between 94 % and 109 %.

All measurements were performed on five replicates of each species and soil sample per sampling site and the mean values were reported. The element content was measured in mg kg^−1^ dry weight and the detection limits of the analyzed elements were as follows (in mg kg^−1^): As 0.005, B 0.0003, Cd 0.0002, Cr 0.001, Cu 0.001, Ni 0.0003, Pb 0.004, and Zn 0.006.

### 2.5 Data analyses

For assessing the enrichment of soils with investigated PTEs, the Enfichment Factor (EF) was calculated as follows:

$$EF=\frac{\left(C_{x}/C_{Mn}\right)\;soil}{\left(C_{x}/C_{Mn}\right)\;background}$$

where $\left(C_{x}/C_{Mn}\right)\;soil$ is ratio between concentrations of potentially enriched element $C_{x}$ and concentration of Mn ($C_{Mn})$ in soil samples, and $\left(C_{x}/C_{Mn}\right)\;background$ is ratio of referenced background values. In this study, Mn was used as a reference element as it is mostly of lithological origin and is subject to minor anthropogenic influences [28]. The background values used for Mn and other investigated PTEs were previously published in the study by Marković et al. [23]. The background value for B was not available, therefore this element is excluded from the calculations. Five contamination categories are distinguished based on the Enrichment Factor [32]: EF < 2 is inadequate to minimal enrichment; EF 2–5 is moderate enrichment; EF 5–20 is significant enrichment; EF 20–40 is very high enrichment; EF > 40 is an extremely high enrichment.

The following calculated coefficients and factors play a crucial role in assessing the potential of plant species for the accumulation and remediation of potentially toxic elements (PTEs).

Enrichment Coefficients for Roots (ECR): evaluates the ratio between the element contents in plant roots and the total content of elements in the soil [33]. If the ECR is greater than 1 and the translocation factor (TF) is less than 1, the plant is considered an excluder, indicating a higher metal accumulation in the roots alone [34].

Enrichment Coefficients for Leaves (ECL): The ECL measures the ability of a plant to accumulate PTEs directly from the soil by comparing the content of elements in the plant leaves with that in the soil [33]. If the ECS is greater than 1, the plant is considered to have phytoextraction potential [35].

Translocation factor (TLF): This factor evaluates the efficiency of element uptake in above-ground plant parts and the transfer capacity of elements from the roots to the leaves [36]. A TLF greater than 1 indicates that the plant has the potential for phytoextraction, which indicates an efficient transfer of elements from the roots to the aerial parts [37].

Metal accumulation index (MAI): Evaluates the overall performance of the examined plants in terms of element accumulation in leaves [38]. The MAI was calculated as:

$$\text{MAI}=\left(1/N\right)\;\sum_{j=1}^{N}Ij$$

where N is the number of elements analyzed and Ij = x/dx is the subindex for variable j obtained by dividing the mean (x) of each metal by its standard deviation (dx). In this particular case, the MAI was calculated as follows:

MAI = (IAs + IB + ICd + ICr + ICu + INi + IPb + IZn)/8 = (xAs/dAs + xB/dB + xCd/dCd + xCr/dCr + xCu/dCu + xNi/dNi + xPb/dPb + xZn/dZn)/8.

The MAI was calculated for each plant at each sampling site and presented as the average MAI value for every plant species.

Comprehensive bio-concentration index (CBCI): Assess the bioaccumulation ability of plants [39]. CBCI was calculated as:

$$CBCI=\left(1/N\right)\sum_{i=1}^{N}\mu_{i}$$

where N is the number of investigated PTEs, and μ is calculated as:

$$\mu \left(x\right)=\left\{ \begin{array}{c} \frac{\begin{array}{c} 0\\ x-x_{\min} \end{array}}{x_{\max}-x_{\min}}\\ 1 \end{array}\right.\;\begin{array}{c} x=x_{\min}\\ x_{\min}<x<x_{\max}\\ x=x_{\max} \end{array}$$

x is the ECL for certain PTE, x_min_ is the minimum value of the ECL of the PTEs among the investigated species, and x_max_ is the maximum ECL of the investigated PTEs among the species. The maximum μ(x) is denoted as the fuzzy value 1, which has contributed most to the comprehensive accumulation ability of species for various PTEs. The minimum is denoted as the fuzzy membership value 0, which has contributed the least to the comprehensive accumulation ability of investigated species.

These indices provide a comprehensive understanding of how efficiently a plant accumulates, excludes or transfers potentially toxic elements, which is crucial for determining its suitability for phytoremediation processes.

The relationship between the content of the investigated elements in the soil, root and leaf samples was evaluated using the non-parametric Spearman correlation (the data do not have a normal distribution). The statistical significance level of the correlations is marked with * for p < 0.05, ** for p < 0.01, and *** for p < 0.001.

Descriptive and multivariate statistical analyzes were performed using Statistica 12.0 [40] and OriginPro 2023b [41] software. The attached map was created with the ArcGis program ArcMap 10.6.1 [42].

## 3 Results and discussion

### 3.1 Soil physical and chemical properties analyses

The physical and chemical properties of the analyzed soils in the riparian zone of the Sava River are summarized in Table 2. Soil pH is an important factor influencing the availability of chemical elements for plants. The pH ranged from 7.16 to 7.68, which classifies the analyzed topsoils as neutral and slightly alkaline according to the Soil Survey Manual [30]. In general, the ability of soil to bind most chemical elements increases with increasing pH and reaches a maximum in the neutral range. Exceptions are As, B and Cr, which are more mobile under alkaline conditions [43]. Most PTEs are also most readily available in soils with an acidic reaction [44]. On the other hand, a pH > of 7 increases the risk of deficiency of essential micronutrients, including B and Zn [43,45].

**Table 2:** Analyzed soil physical and chemical properties.

Sampling site	pH (H_2_O)	OM (%)	Coarse sand (2–0.2 mm; %)	Fine sand (0.2–0.06 mm; %)	Silt (%)	Clay (%)	Soil texture class
MOJ	7.16	19.43	44.77	13.77	37.26	4.20	Sandy loam
RAD	7.42	1.75	28.81	50.43	16.75	4.01	Loamy sand
LIT	7.61	1.1	55.46	34.63	9.29	0.62	Sand
VRH	7.54	2.0	13.42	56.57	25.69	4.32	Sandy loam
CAT	7.68	1.77	3.81	51.53	37.94	6.72	Sandy loam
ZAG	7.42	0.36	30.83	45.92	18.41	4.84	Loamy sand

The amount of organic matter (OM) is an important characteristic as it controls several other soil properties by increasing soil aeration and water capacity, while the binding of chemical elements reduces their bioavailability [43,44,46]. Organic matter strongly binds Cr and Pb, while elements such as Zn are only weakly bound [44]. The percentage of organic matter in the soils studied was generally <2, except in MOJ where the percentage of OM was 19.43 % (Table 2). These results indicate possible availability of analyzed PTEs in investigated soils.

The granulometric composition affects all other physical and chemical properties of the soil. The soils analyzed have a higher proportion of sand particles, which is why they are classified as sand, sandy loam and loamy sand (Table 2). A higher proportion of sand is a common feature of the riparian soils [47]. In sandy soils, soil particles, organic matter, minerals and other chemical elements are more easily washed away by rainfall and floods [48].

**Fig. 2:**
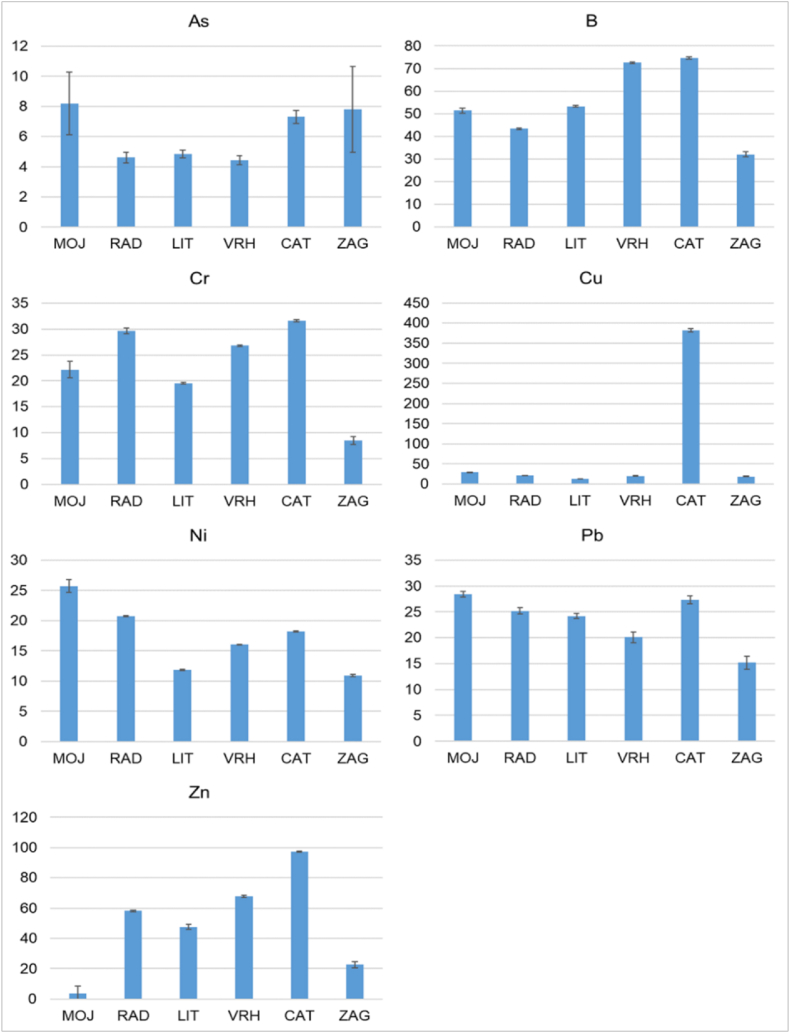
PTE content in soils presented as average values with standard deviation, in mg kg^−1^.

The analysis of the total content of chemical elements in the soils at the investigated sites included the following elements: As, B, Cd, Cr, Cu, Ni, Pb and Zn, and the reuslts are presented in Fig. 2. Cd was not detected in any of the soil samples analyzed. Several guidelines were used to assess the level of the soil contamination: the natural background of the study area [23], the average element content in the Foregs Geochemical Atlas of Europe [49], and the critical range of elements for plants [50].

Most of the PTEs measured were below the values specified in the guidelines. The exceptions are measured contents of Cu and Zn. The measured Cu content in soils varied from 12.66 mg kg^−1^ at the LIT sampling site to 382.26 mg kg^−1^ at the CAT (Fig. 2). The content of Cu measured at CAT was several times higher than the natural background of the study area (24.12 mg kg^−1^) [23], the average element content in European soils (17.3 mg kg^−1^) [49], and above the critical range of elements for plants (60–125 mg kg^−1^) [50]. The Zn content in the soils ranged from 3.67 mg kg^−1^ at the MOJ, to 97.30 mg kg^−1^ at the CAT sampling site. The values measured at CAT were slightly higher than the natural background of the study area (91.64 mg kg^−1^) [23], the average Zn content in European soils (68.10 mg kg^−1^) [49], and within the critical range of Zn for plants (70–400 mg kg^−1^) [50].

In addition to the guidelines, the level of soil contamination was also assessed using the Enrichment factor (EF). The results are presented at Table 3.

**Table 3:**
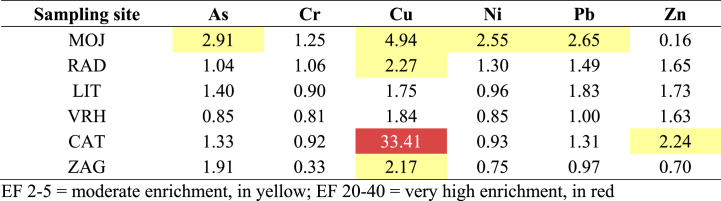
Enrichment factor in investigated soils.

According to the EF, moderate enrichment (EF 2–5) was found in MOJ for As, Cu, Ni and Pb, in RAD and ZAG for Cu and in CAT for Zn. At the CAT sampling site, the enrichment of the soil with Cu was in the range of a very high enrichment (EF 20–40) [32].

A moderate enrichment of As, Cu, Ni and Pb in MOJ is to be expected due to the rich mining history of this town. In the period between the founding of the settlement and the end of the Second World War, the population was engaged in the mining and processing of iron ore and the production of cement. Today, there are no longer any active industrial plants [51]. Considering the fact that the parent rock is rich in minerals and the additional anthropogenic impact of mining, it could be concluded that the sources and enrichment of PTEs in the soil are of both natural and anthropogenic in origin. The Cu and Zn enrichment at the RAD and ZAG sampling sites is most likely due to industrial activities. Upstream of RAD, there are metallurgical and wood processing industries, which are the main anthropogenic sources of pollution in the area [51]. The most important industries in the ZAG area are the manufacture of electrical appliances, the chemical and pharmaceutical industries, the textile industry and food processing [51].

The enrichment of the soil with Cu at CAT is most likely anthropogenic. This site is located in the "Posavska" vineyard, where the application of fungicides, which are Cu compounds, is very common, leading to an accumulation of Cu in the soil and thus to soil contamination and potential toxicity to plants [52,53].

### 3.2 PTE content in plant samples

The measured PTE contents in roots and leaves of *Solidago canadensis, Reynoutria japonica* and *Impatiens glandulifera* are shown in Table 4, Table 5. The average amount of elements in the roots of *S. canadensis* followed this order: Zn > Cu > B > Cr > Pb > Ni > As > Cd, while in the leaves the order was as follows: B > Zn > Cu > Pb > Cr > As ≈ Ni > Cd. In the roots of *R. japonica*, the average accumulation of the elements was as follows: Zn > B > Cu > Cr > Pb > Ni > As > Cd, while the order in the leaves was: B > Zn > Cu > Pb > Cr > As > Ni=Cd. The roots of *I. glandulifera* accumulated PTEs in this order: B > Zn > Cu > Pb > Cr > Ni > As > Cd, while the leaves accumulated Zn > B > Cu > Pb > Cr > Ni > As > Cd.

**Table 4:** PTE content in roots, presented as mean ± standard deviation, in mg kg^−1^.

Site	Species	As	B	Cd	Cr	Cu	Ni	Pb	Zn
MOJ	*S. canadensis*	1.12 ±0.89	21.08 ±0.90	0.25 ±0.00	2.92 ±0.46	15.87 ±1.18	2.00 ±0.16	2.33 ±0.26	17.04 ±3.93
	*R. japonica*	<DL	12.21 ±0.85	0.25 ±0.00	2.21 ±0.46	9.04 ±1.02	0.50 ±0.16	1.29 ±0.58	19.13 ±1.72
	*I. glandulifera*	1.33 ±0.10	14.75 ±0.42	0.47 ±0.07	2.50 ±0.21	2.11 ±0.38	0.47 ±0.13	3.56 ±0.23	21.95 ±0.66
RAD	*S. canadensis*	<DL	11.00 ±1.08	0.17 ±0.00	2.61 ±0.56	13.75 ±0.65	0.28 ±0.25	1.33 ±0.35	21.11 ±0.68
	*R. japonica*	<DL	11.22 ±0.82	<DL	1.67 ±0.39	6.11 ±0.46	1.67 ±0.15	1.28 ±0.34	12.53 ±0.63
	*I. glandulifera*	1.44 ±	24.12 ±1.78	0.44 ±0.09	7.33 ±0.65	6.50 ±0.65	3.25 ±0.39	11.63 ±0.98	22.07 ±2.95
LIT	*S. canadensis*	<DL	22.11 ±2.12	0.28 ±0.09	21.80 ±3.93	21.25 ±0.48	5.55 ±1.08	7.14 ±1.03	27.05 ±0.49
	*R. japonica*	<DL	11.39 ±1.29	0.06 ±0.09	2.42 ±0.76	5.78 ±0.46	0.86 ±0.32	2.47 ±0.49	7.05 ±0.84
	*I. glandulifera*	<DL	12.78 ±1.15	0.22 ±0.09	2.36 ±0.44	4.14 ±0.32	0.50 ±0.33	1.92 ±0.38	17.53 ±1.11
VRH	*S. canadensis*	<DL	16.50 ±1.06	0.14 ±0.07	10.14 ±1.98	19.94 ±0.84	2.47 ±0.51	3.36 ±0.72	28.80 ±1.52
	*R. japonica*	<DL	10.19 ±0.49	0.03 ±0.00	1.58 ±0.09	7.08 ±0.33	0.64 ±0.13	1.75 ±0.31	10.86 ±0.40
	*I. glandulifera*	<DL	16.23 ±0.61	0.06 ±0.00	4.11 ±0.78	4.00 ±0.40	1.00 ±0.30	4.67 ±0.21	16.87 ±1.01
CAT	*S. canadensis*	<DL	13.96 ±0.79	0.06 ±0.01	4.52 ±0.80	16.88 ±1.21	0.92 ±0.17	1.78 ±0.46	20.79 ±1.65
	*R. japonica*	<DL	10.71 ±0.86	<DL	3.72 ±0.09	4.08 ±0.20	1.05 ±0.09	1.25 ±0.17	12.13 ±0.67
	*I. glandulifera*	<DL	15.67 ±1.06	0.11 ±0.03	1.78 ±0.06	3.75 ±0.35	0.53 ±0.22	1.06 ±0.60	15.44 ±0.96
ZAG	*S. canadensis*	<DL	12.69 ±0.47	<DL	4.28 ±0.37	22.72 ±0.29	0.81 ±0.16	1.61 ±0.17	17.80 ±0.50
	*R. japonica*	0.92 ±	9.28 ±0.47	<DL	6.03 ±0.30	7.36 ±1.28	2.25 ±0.22	0.67 ±0.34	18.72 ±0.97
	*I. glandulifera*	<DL	22.10 ±1.20	0.36 ±0.07	4.33 ±0.11	7.52 ±0.55	2.72 ±0.27	4.05 ±0.40	2.53 ±0.44
Deficit		/	5–30^b^	/	/	2–5^b^	/	/	10–20^b^
Optimum range		1–1.7^b^	10–100^b^	0.002–1^b^	0.1–0.5^b^	5–30^b,c^	0.1-5^a,b^	0.2–10^b,c^	27–150^b^
Toxic range		5–10^a.b^	50–200^b^	5–30^a,b^	5–30^a,b^	20–100^b,c^	10–100^a,b^	30–300^a,b^	100–400^a,b^

Deficit, optimum and toxic range values: a Alloway [50]; b Kabata-Pendias [44]; c Pugh et al. [54]; <DL – below the detection limit.

**Table 5:** PTE content in leaves, presented as mean ± standard deviation, in mg kg^−1^.

Site	Species	As	B	Cd	Cr	Cu	Ni	Pb	Zn
MOJ	*S. canadensis*	1.17 ±0.29	45.16 ±0.41	<DL	1.50 ±0.59	8.00 ±0.45	0.33 ±0.03	<DL	17.20 ±2.90
	*R. japonica*	0.39 ±0.06	36.88 ±0.60	<DL	0.47 ±0.16	4.83 ±0.45	<DL	1.25 ±0.25	13.43 ±1.57
	*I. glandulifera*	0.33 ±0.02	35.02 ±0.33	0.50 ±0.00	1.08 ±0.09	12.50 ±0.21	1.00 ±0.00	1.47 ±0.31	53.36 ±1.21
RAD	*S. canadensis*	1.11 ±0.25	47.08 ±0.34	<DL	0.50 ±0.15	5.58 ±0.42	0.03 ±0.00	1.67 ±0.18	28.07 ±2.58
	*R. japonica*	<DL	29.45 ±0.07	<DL	0.22 ±0.09	2.36 ±0.13	<DL	1.08 ±0.27	19.92 ±0.08
	*I. glandulifera*	0.36 ±0.05	23.36 ±0.30	0.50 ±0.00	0.75 ±0.09	7.64 ±0.12	<DL	1.64 ±0.29	61.30 ±1.00
LIT	*S. canadensis*	1.50 ±0.14	52.12 ±0.87	0.22 ±0.09	5.66 ±0.15	9.25 ±0.17	2.22 ±0.25	5.61 ±1.58	13.76 ±1.43
	*R. japonica*	<DL	34.29 ±0.48	<DL	0.42 ±0.09	3.81 ±0.69	<DL	0.69 ±0.39	6.02 ±0.34
	*I. glandulifera*	<DL	20.36 ±0.42	0.17 ±0.00	1.22 ±0.23	14.22 ±0.42	<DL	1.86 ±0.36	58.59 ±2.63
VRH	*S. canadensis*	0.44 ±0.07	42.75 ±1.51	<DL	1.11 ±0.14	12.30 ±0.59	0.31 ±0.02	1.69 ±0.32	38.84 ±2.67
	*R. japonica*	<DL	38.86 ±1.02	<DL	0.56 ±0.09	3.22 ±0.62	<DL	1.17 ±0.30	4.74 ±1.02
	*I. glandulifera*	1.44 ±0.38	28.48 ±0.56	0.50 ±0.00	2.42 ±0.27	14.60 ±0.38	1.53 ±0.12	2.97 ±0.37	68.29 ±2.72
CAT	*S. canadensis*	0.53 ±0.09	36.86 ±0.99	<DL	1.75 ±0.25	8.16 ±0.21	0.22 ±0.04	2.14 ±0.07	21.14 ±2.35
	*R. japonica*	<DL	33.14 ±0.39	<DL	1.17 ±0.15	2.19 ±0.51	<DL	1.58 ±0.27	0.88 ±0.13
	*I. glandulifera*	0.89 ±0.07	25.64 ±1.54	0.39 ±0.09	0.36 ±0.13	14.83 ±0.39	0.58 ±0.09	0.14 ±0.04	43.28 ±2.51
ZAG	*S. canadensis*	0.39 ±0.06	37.61 ±0.32	0.22 ±0.09	5.44 ±0.43	10.39 ±0.71	2.00 ±0.26	5.11 ±0.14	12.10 ±0.81
	*R. japonica*	<DL	23.43 ±0.27	<DL	0.33 ±0.00	2.11 ±0.28	<DL	2.16 ±0.30	17.44 ±0.71
	*I. glandulifera*	0.53 ±0.11	32.67 ±0.94	0.50 ±0.00	2.31 ±0.20	11.58 ±0.55	1.50 ±0.18	2.11 ±0.27	26.36 ±0.89
Deficit		/	5–30^b^	/	/	2–5^b^	/	/	10–20^b^
Optimum range		1–1.7^b^	10–100^b^	0.002–1^b^	0.1–0.5^b^	5–30^b,c^	0.1-5^a,b^	0.2–10^b,c^	27–150^b^
Toxic range		5–10^a.b^	50–200^b^	5–30^a,b^	5–30^a,b^	20–100^b,c^	10–100^a,b^	30–300^a,b^	100–400^a,b^

Deficit, optimum and toxic range values: Alloway [50]; b Kabata-Pendias [44]; c Pugh et al. [54]; <DL – below the detection limit.

The guidelines proposed by Alloway [55] and Kabata-Pendias [44] were used to evaluate the uptake and accumulation of PTEs in the analyzed plants and to determine the areas of deficit, optimum or toxicity to plants for each of the studied PTEs.

All investigated species accumulated As, Cd and Pb in their roots and leaves in the optimal range or below the optimum of these elements in plants (Table 4, Table 5). Most of the plants studied accumulated B in roots and leaves in a range considered normal for plants. The exceptions are *R. japonica*, which accumulated 9.28 mg kg^−1^ in the roots at the ZAG sampling site, which can be considered deficient, and *S. canadensis*, which accumulated 52.12 mg kg^−1^ in the leaves at the LIT sampling site, which could be considered toxic. *Solidago canadensis* accumulated Cr in its roots (at the LIT and VRH sampling sites) and leaves (at the LIT and ZAG sampling sites) in a range that could be considered toxic (Table 4, Table 5), while *R. japonica* accumulated this element in its roots at the ZAG sampling site in a potentially toxic range (Table 4). Potentially toxic amounts of Cu were accumulated in the roots of *S. canadensis* at the LIT and ZAG sampling sites, as was Ni in the roots of this species at the LIT sampling site.

At the same time, the deficit of the essential elements was measured. Namely, *I. glandulifera* accumulated Cu in a range that can be considered a deficit in its roots at the MOJ, LIT and CAT sampling sites. *Reynoutria japonica* also accumulated Cu in the area of deficit in its roots at the CAT sampling site and in its leaves at all sampling sites. This species also accumulated Zn in the deficit area in the roots and leaves at all sampling sites. A potential Zn deficit was also detected in the roots of *I. glandulifera* at the LIT, VRH, CAT and ZAG sites, while *S. canadensis* accumulated this element in the range of deficit in its roots (at the MOJ and ZAG sites) and leaves (at the MOJ, LIT and ZAG sites).

The low accumulation of essential elements such as Cu and Zn could most likely be due to the alkaline pH of the soil and the high proportion of sand in the soil structure (Table 2).

The ability of IPS such as *Solidago canadensis* to tolerate or avoid PTEs is a remarkable aspect of their ecological adaptation [56]. Previous studies have shown that *S. canadensis* accumulates similar levels of Zn in its roots and leaves in agricultural areas of Poland [57]. The same authors have shown that this species accumulates much higher amounts of Pb and Zn in industrial areas, compared to results obtained in this study. In areas with different pollution levels and sources in Poland, Dambiec et al. [58] measured similar content of Cd, Cu, Ni and Pb in roots and leaves of *S. canadensis*, while the content of Zn was much higher than obtained in this study. The same was found in the leaves of this species growing on municipal solid waste landfills [59]. These differences could be due to the different conditions at the sampling sites (e.g. soil properties and PTE content in the soil), as well as the methods used to determine the PTE content in plant samples.

The evaluation of the PTE content of *Reynoutria japonica* is very important in view of its use in traditional Chinese medicine and the new research results showing the importance of this species in the treatment of cardiovascular and digestive diseases [60]. In the urban areas of the city of Zagreb, previous studies by Hulina and Ðumija [61] have shown that the accumulation of Cd, Cu, Pb and Zn in the leaves of *R. japonica* is higher compared to the values measured in this study at the ZAG site. These differences could be due to the different methodology used to assess the PTE content.

In the existing literature, there are also differences in the accumulation of PTEs in *Reynoutria* species depending on the sampling site level of the pollution. The content of Cd, Cu, Ni and Pb measured in the rhizomes and leaves of *R. japonica* in the present study were very similar to those measured in the study by Lerch et al. [62] at the PTE-clean control site. On the other hand, the accumulation of Cr, Cu, Pb in the leaves of *R. japonica* growing at sites with different levels of disturbance in Poland [63] was similar to the results of this research, while we measured much lower values for Cd, Ni and Zn. Similarly, Berchová-Bímová et al. [56] measured higher levels of Cd and Pb in the rhizome and leaves of *R. japonica, R. sachalinensis* and *Reynoutria x bohemica* at sites with different pollution sources in Prague, compared to the results obtained in this study. Moreover, previous studies have shown that *Reynoutria x bohemica* is effective in accumulation of PTEs, especially Cr and Ni, in urban areas [64]. At highly polluted sites caused by metallurgical industries, *R. japonica* accumulated very high levels of Cd, Cu, Pb and Zn [65], compared to contents obtained in this study. Experimental studies have shown that this species is capable of accumulating extremely high contents of Pb [66], Cr and Zn [13].

Species of the genus *Impatiens* have shown a high tolerance and accumulation potential for Cd and Pb, which represents a potential for phytoremediation of these elements [67]. For example, the experimental study on *I. glandulifera* has shown that it is able to accumulate over 1000 mg kg^−1^ Cd in its roots [68]. *Impatiens walleriana* also accumulates Cd in the order of 100 mg kg^−1^ and is considered a Cd hyperaccumulator [69,70]. The same species accumulated similar amounts of Cr and Zn in roots and leaves in urban areas of Brazil [71], as measured in the present study. An experimental study on *I. balsamina* has shown that this species accumulates similar amounts of Ni in the roots [72] as measured in the present study.

**Fig. 3:**
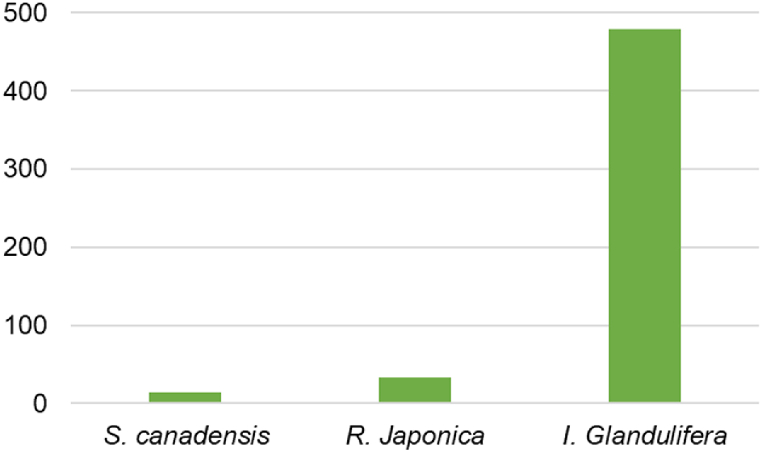
MAI index.

Overall accumulation of PTE in the leaves of selected plants was evaluated using the metal accumulation index (MAI) (Fig. 3). The results showed that *Impatiens glandulifera* had the highest potential for accumulation of the studied PTEs (MAI = 479), while the potential for PTE accumulation was significantly lower in *R. japonica* (MAI = 32.97) and *S. canadensis* (MAI = 14.5).

### 3.3 The ability of plants for phytoremediation and biomonitoring of soils

The use of enrichment coefficients for roots (ECR), leaves (ECL) and translocation factors (TLF) is a common approach to analyze and evaluate the potential of plant species for phytoremediation and biomonitoring of contaminants, especially PTEs. These coefficients provide valuable insights into the ability of plant species to accumulate, exclude or translocate certain elements, which is crucial for understanding their potential role in phytoremediation and biomonitoring. By comparing these coefficients between different plant species and sampling sites, it would be possible to determine which species is more effective in combating soil contamination and monitoring pollutant levels [73].

*Solidago canadensis* showed an ECR >1 for Cr at the LIT, Cu at the LIT and ZAG and Zn at the MOJ sampling site (Table 6). At the same time, the TFs for Cr and Cu at these sampling sites are lower than 1, suggesting that this species acts as a Cr and Cu excluder. The ECS was higher than 1 for B in RAD and ZAG, and for Zn in MOJ. Together with TF > 1 for these elements, the results indicate the potential of *S. canadensis* for phytoextraction of B and Zn. Bielecka and Królak [57] found similar ECR and TLF values for Pb and Zn in agricultural areas, while these factors were lower in the industrialized areas of Poland. On the other hand, Gworek et al. [59] found higher ECLs for Cd, Cu, Ni and Zn at municipal solid waste landfills, while ECL for Cr and Pb were similar to the values determined in the present study.

**Table 6:** ECR, ECS and TFL coefficients for the studied species.

ECR																								
*Solidago canadensis*									*Reynoutria japonica*								*Impatiens glandulifera*							
	As	B	Cd	Cr	Cu	Ni	Pb	Zn	As	B	Cd	Cr	Cu	Ni	Pb	Zn	As	B	Cd	Cr	Cu	Ni	Pb	Zn
MOJ	0.14	0.41	/	0.13	0.55	0.08	0.08	**4.65**	/	0.24	/	0.10	0.31	0.02	0.05	**5.22**	0.16	0.29	/	0.11	0.07	0.02	0.13	**5.99**
RAD	/	0.25	/	0.09	0.65	0.01	0.05	0.36	/	0.26	/	0.06	0.29	0.08	0.05	0.21	0.31	0.56	/	0.25	0.31	0.16	0.46	0.38
LIT	/	0.42	/	**1.12**	**1.68**	0.47	0.30	0.57	/	0.21	/	0.19	0.46	0.07	0.10	0.15	0.00	0.24	/	0.12	0.33	0.04	0.08	0.37
VRH	/	0.23	/	0.38	0.99	0.15	0.17	0.43	/	0.14	/	0.06	0.35	0.04	0.09	0.16	0.00	0.22	/	0.15	0.20	0.06	0.23	0.25
CAT	/	0.19	/	0.14	0.04	0.05	0.06	0.21	/	0.14	/	0.12	0.01	0.06	0.05	0.12	0.00	0.21	/	0.06	0.01	0.03	0.04	0.16
ZAG	/	0.40	/	0.50	**1.23**	0.07	0.11	0.79	0.12	0.29	/	0.71	0.40	0.21	0.04	0.83	0.00	0.69	/	0.51	0.41	0.25	0.27	0.11
ECL																								

*Solidago canadensis*									*Reynoutria japonica*								*Impatiens glandulifera*							

	As	B	Cd	Cr	Cu	Ni	Pb	Zn	As	B	Cd	Cr	Cu	Ni	Pb	Zn	As	B	Cd	Cr	Cu	Ni	Pb	Zn

MOJ	0.14	0.88	/	0.07	0.28	0.01	0.00	**4.69**	0.05	0.72	/	0.02	0.17	/	0.04	**3.66**	0.04	0.68	/	0.05	0.43	0.04	0.05	**14.56**
RAD	0.24	**1.09**	/	0.02	0.26	/	0.07	0.48	0.00	0.68	/	0.01	0.11	/	0.04	0.34	0.08	0.54	/	0.03	0.36	/	0.07	**1.05**
LIT	0.31	0.98	/	0.29	0.73	0.19	0.23	0.29	0.00	0.64	/	0.02	0.30	/	0.03	0.13	0.00	0.38	/	0.06	**1.12**	/	0.08	**1.23**
VRH	0.10	0.59	/	0.04	0.61	0.02	0.08	0.57	0.00	0.54	/	0.02	0.16	/	0.06	0.07	0.32	0.39	/	0.09	0.73	0.10	0.15	**1.01**
CAT	0.07	0.49	/	0.06	0.02	0.01	0.08	0.22	0.00	0.44	/	0.04	0.01	/	0.06	0.01	0.12	0.34	/	0.01	0.04	0.03	0.01	0.44
ZAG	0.05	**1.17**	/	0.64	0.56	0.18	0.34	0.53	0.00	0.73	/	0.04	0.11	/	0.14	0.77	0.07	**1.02**	/	0.27	0.63	0.14	0.14	**1.17**
TLF																								

*Solidago canadensis*									*Reynoutria japonica*								*Impatiens glandulifera*							

	As	B	Cd	Cr	Cu	Ni	Pb	Zn	As	B	Cd	Cr	Cu	Ni	Pb	Zn	As	B	Cd	Cr	Cu	Ni	Pb	Zn

MOJ	**1.04**	**2.14**	/	0.51	0.50	0.17	0.00	**1.01**	0.00	**3.02**	/	0.21	0.53	/	0.97	0.70	0.25	**2.37**	**1.06**	0.43	**5.92**	**2.12**	0.41	**2.43**
RAD	/	**4.28**	/	0.19	0.41	0.10	**1.25**	**1.33**	0.00	**2.62**	/	0.13	0.39	/	0.85	**1.59**	0.25	0.97	**1.13**	0.10	**1.18**	/	0.14	**2.78**
LIT	/	**2.36**	/	0.26	0.44	0.40	0.79	0.51	0.00	**3.01**	/	0.17	0.66	/	0.28	0.85	0.00	**1.59**	0.75	0.52	**3.44**	/	0.97	**3.34**
VRH	/	**2.59**	/	0.11	0.62	0.12	0.50	**1.35**	0.00	**3.81**	/	0.35	0.45	/	0.67	0.44	0.00	**1.76**	**8.99**	0.59	**3.65**	**1.53**	0.64	**4.05**
CAT	/	**2.64**	/	0.39	0.48	0.24	**1.20**	**1.02**	0.00	**3.09**	/	0.31	0.54	/	**1.27**	0.07	0.00	**1.64**	**3.50**	0.20	**3.96**	**1.11**	0.13	**2.80**
ZAG	/	**2.96**	/	**1.27**	0.46	**2.48**	**3.17**	0.68	0.00	**2.52**	/	0.06	0.29	/	**3.25**	0.93	0.00	**1.48**	**1.39**	0.53	**1.54**	0.55	0.52	**10.44**

Values higher than 1 are denoted in bold.

*Reynoutria japonica* has an ECR and ECL higher than 1 for Zn at the ZAG sampling site. At the same time, the TLF for Zn at the ZAG was lower than 1, indicating that this species acts as an excluder of Zn at this site (Table 6). TLF >1 was found for B at all sampling sites, for Pb at CAT and ZAG and for Zn at the RAD sampling site. These results indicate some potential for phytoextraction of these elements, especially of B. Lerch et al. [62] found higher ECR and TLF values for Cd and Ni, while these indices were similar for Cu and Zn at both the polluted and control sites. Vidican et al. [65] found similar ECR, ECL and TLF values for Cu, Pb and Zn at heavily polluted sites caused by the metallurgical industry as calculated in the present study. These studies confirm the potential of *R. japonica* for the translocation of Pb and Zn.

In *Impatiens glandulifera*, ECR>1 for Zn was found at sampling site MOJ, while ECL>1 for B was found at ZAG, Cu at LIT and Zn at all sampling sites except CAT (Table 6). Translocation higher than 1 was found at all sampling sites for Cu and Zn, while TLF>1 was found for B and Cd at all sampling sites except RAD and LIT respectively (Table 6). TLF>1 were also found for Ni at MOJ, VRH and CAT sampling sites. These factors indicate the ability of this species to phytoextract B, Cd, Cu and Zn. Coakley et al. [68] also found ECR and TLF values higher than 1 for Cd in *Impatiens glandulifera* under experimental conditions. Similarly, the ECR and TLF values for Cd in *I. walleriana* were higher than 1, indicating the good suitability of this species for phytoremediation of Cd-polluted soils [74]. Similar ECR and TLF values for Ni as in this study were determined in the experimental study by Yasin et al. [72] in *I. balsamina*.

**Fig. 4:**
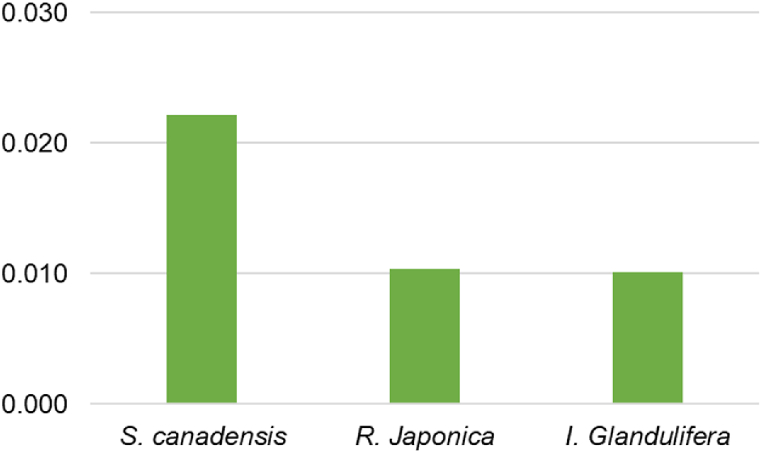
CBCI index.

The general ability of plants to bioaccumulate PTE was also investigated using the comprehensive bio-concentration index (CBCI) (Fig. 4). The results show that *R. japonica* and *I. glandulifera* have a similar ability to accumulate PTEs from the soil in their leaves (CBCI = 0.010), while *S. canadensis* is more efficient in PTE accumulation in leaves (CBCI = 0.022).

**Fig. 5:**
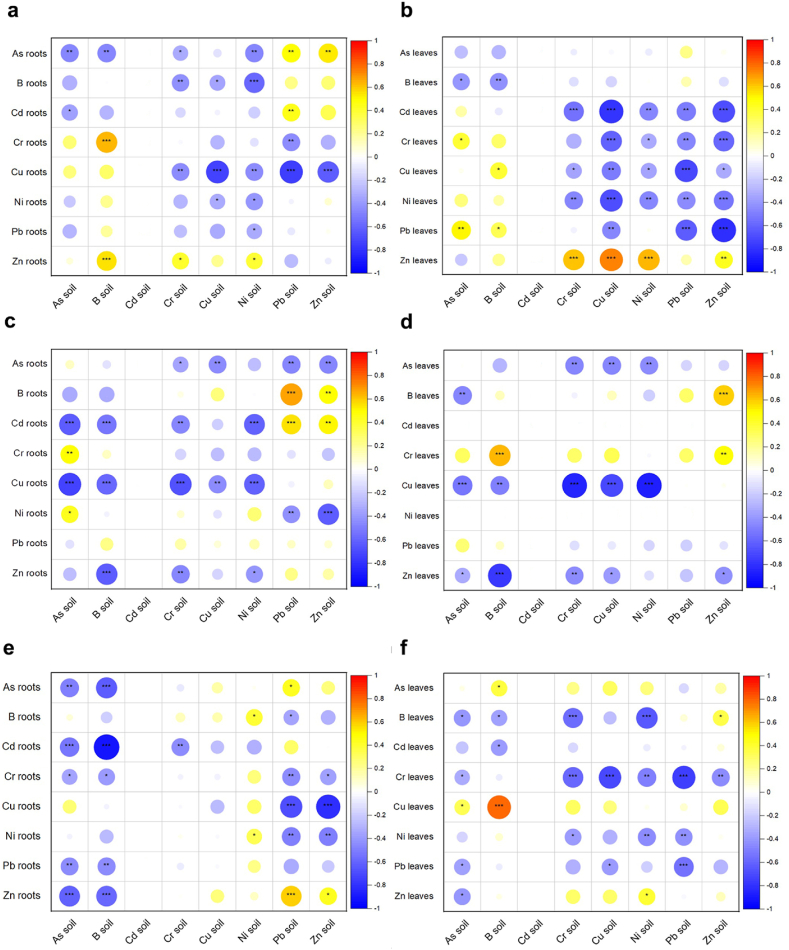
Spearman correlations of PTE contents in: a) *Solidago canadensis* roots and soil; b) *Solidago canadensis* leaves and soil; c) *Reynoutria japonica* roots and soil; d) *Reynoutria japonica* leaves and soil; e) *Impatiens glandulifera* roots and soil; f) *Impatiens glandulifera* leaves and soil; * for p < 0.05, ** for p < 0.01, and *** for p < 0.001.

The ability of analyzed plants to bioindicate PTEs in soils was evaluated by Spearman correlations of PTE content in plants and soils, and the results are shown in Fig. 5. Most of the obtained correlations were significantly negative, indicating that the analyzed species are not suitable for bioindication of examined PTEs in riparian soils.

## 4 Conclusion

This study provides valuable information on the accumulation of PTE in leaves and roots of *Solidago canadensis, Reynoutria japonica* and *Impatiens glandulifera*, invasive species in the Sava river basin. The study showed that the soils in the upper reaches of the Sava River are neutral to slightly alkaline, have a low organic matter content and a high proportion of sand in the granulometric composition. The soils contain higher levels of Cu and Zn, and the enrichment factor revealed a moderate to very high enrichment of the soils with Cu. At individual sampling sites, the plants studied accumulated B, Cr, Cu and Ni in amounts that can be considered toxic to plants and at the same time a deficit of essential Cu and Zn was observed. The metal accumulation index showed that *Impatiens glandulifera* has the highest capacity for the uptake of PTE in the leaves. According to ECR, ECL and TLF, *Solidago canadensis* has been shown to exclude Cr and Cu, while it has a potential for phytoextraction of B and Zn. *Reynoutria japonica* has some potential for phytoextraction of Pb, Zn and especially B, while *Impatiens glandulifera* has the ability for phytoextraction of B, Cd, Cu and Zn. The CBCI index showed that *S. canadensis* has the highest bioaccumulation potential. The analyzes showed that none of the investigated species is suitable for bioindication of the investigated PTEs in the riparian soils of the Sava River. Considering the higher levels of Cu and Zn of the Sava River riparian soils, *Impatiens glandulifera* is the most suitable species for phytoremediation of these elements among the investigated species. Extensive research is still needed to find suitable plants for the bioindication of PTEs in the soils upstream of the Sava River. In addition, future research could also focus on studying the effects of accumulated PTEs on the ecophysiological properties of the plants themselves.

## Data availability statement

The data obtrained in this study will be available on request.

## Submission declaration

This article is original and has not been previously published, in whole or in part. This work is not under consideration by any other journal elsewhere and its publication is approved by all authors. In case of the acceptance of this paper, it will not be published elsewhere in the same form, in English or in any other language, including electronically without the written consent of the copyright-holder.

## CRediT authorship contribution statement

**Zorana Miletić:** Writing – review & editing, Writing – original draft, Conceptualization. **Snežana Jarić:** Validation, Supervision. **Milica Jonjev:** Methodology, Data curation. **Miroslava Mitrović:** Writing – review & editing, Validation. **Dragana Pavlović:** Software, Investigation. **Marija Matić:** Visualization, Investigation. **Pavle Pavlović:** Resources, Funding acquisition.

## Declaration of competing interest

The authors declare that they have no known competing financial interests or personal relationships that could have appeared to influence the work reported in this paper.
